# Intra-articular injection of *N*-acetylglucosamine and hyaluronic acid combined with PLGA scaffolds for osteochondral repair in rabbits

**DOI:** 10.1371/journal.pone.0209747

**Published:** 2018-12-31

**Authors:** Hsueh-Chun Wang, Yi-Ting Lin, Tzu-Hsiang Lin, Nai-Jen Chang, Chih-Chan Lin, Horng-Chaung Hsu, Ming-Long Yeh

**Affiliations:** 1 Department of Biomedical Engineering, National Cheng Kung University, Tainan, Taiwan; 2 Department of Sports Medicine, Kaohsiung Medical University, Kaohsiung, Taiwan; 3 Laboratory Animal Center, Department of Medical Research, Chi-Mei Medical Center, Tainan, Taiwan; 4 Department of Orthopedics, China Medical University Hospital, Taichung, Taiwan; 5 Medical Device Innovation Center, National Cheng Kung University, Tainan, Taiwan; University Hospital Modena and Reggio Emilia, ITALY

## Abstract

Repairing damaged articular cartilage is particularly challenging because of the limited ability of cartilage to perform self-repair. Intra-articular injections of *N*-acetylglucosamine (GlcNAc) comprise a method of repairing full-thickness articular cartilage defects in the rabbit knee joint model. To date, the effects of administration of GlcNAc and hyaluronic acid (HA) have been investigated only in the context of osteoarthritis treatment. Therefore, we evaluated the therapeutic effects of using cell-free porous poly lactic-co-glycolic acid (PLGA) graft implants and intra-articular injections of GlcNAc or HA in a rabbit model of osteochondral regeneration to investigate whether they have the potential for inducing osteochondral regeneration when used alone or simultaneously. Twenty-four rabbits were randomized into one of four groups: the scaffold-only group (PLGA), the scaffold with intra-articular injections of GlcNAc (PLGA+G) group, twice per week for four weeks; the scaffold with intra-articular injections of HA group (PLGA+HA) group, once per week for three weeks; and the scaffold with intra-articular injections of GlcNAc and HA (PLGA+G+HA) group, once per week for three weeks. Knees were evaluated at 4 and 12 weeks after surgery. At the end of testing, only the PLGA+G+HA group exhibited significant bone reconstruction, chondrocyte clustering, and good interactions with adjacent surfaces at 4 weeks. Additionally, the PLGA+G+HA group demonstrated essentially original hyaline cartilage structures that appeared to have sound chondrocyte orientation, considerable glycosaminoglycan levels, and reconstruction of the bone structure at 12 weeks. Moreover, the PLGA+G+HA group showed organized osteochondral integration and significantly higher bone volume per tissue volume and trabecular thickness. However, there were no significant differences between the PLGA+G and PLGA+HA groups except for gap formation on subchondral bone in the PLGA+G group. This study demonstrated that PLGA implantation combined with intra-articular injections of GlcNAc and HA allowed for cartilage and bone regeneration and significantly promoted osteochondral regeneration in rabbits without supplementation of exogenous growth factors. And the combination of this two supplements with PLGA scaffold could also prolong injection interval and better performance than either of them alone for the reconstruction of osteochondral tissue in the knee joints of rabbits.

## Introduction

Osteochondral defect (OCD), a type of joint disorder that often occurs with disease or repetitive trauma in the bone, cartilage, and bone–cartilage interface [1]. Left untreated, it may progress to degenerative osteoarthritis (OA) with disability and function loss. Current clinical treatments for cartilage repair include hyaluronan injection, microfracture, bone marrow stimulation, mosaicplasty as autologous osteochondral transplantation, and autologous chondrocyte implantation. Nevertheless, problems exist such as donor site morbidity, poor integration with host tissue, fibrocartilage formation, and chondrocyte dedifferentiation [2–5]. Therefore, tissue engineering has emerged and may offer significant advantages compared with traditional clinical treatment methods. Cells, scaffolds, and signals are three important factors involved in tissue engineering. Regarding the scaffold, cell-free and cell-seeded scaffolds are two approaches that are typically used; however, the cell-free method is often adopted due to needless of cell expansion and less time-consumption. Ideal scaffolds should provide mechanical support and guide cell adhesion, proliferation, and/or differentiation to regenerate osteochondral tissue. Poly lactic-co-glycolic acid (PLGA) is a synthetic material and the copolymer of polylactic acid and polyglycolic acid [6]. This implant possesses superior mechanical strength [7] compared to naturally derived materials and provides a provisional matrix for osteochondral regeneration [8, 9]. PLGA is a safe biomaterial for clinical applications [10] that has been approved by the United States Food and Drug Administration [11, 12].

Extracellular matrices in cartilage provide a microenvironment for cells to maintain homeostasis and differentiation properties for specific tissues. Glucosamine (GlcN) and hyaluronic acid (HA) are the main components in extracellular matrices in articular cartilage. Both have been clinically used for OA treatment for several decades, resulting in chondroprotective effects [13] or viscosupplementation [14]. However, the use of GlcN for OA treatment remains controversial because not all trials have shown significant difference in Western Ontario and McMaster Universities Osteoarthritis Index values for pain or function compared with placebo [15–17]. *N*-acetylglucosamine (GlcNAc) is one of three forms of GlcN; it is more stable and is available commercially. Previous in vitro studies have shown that GlcNAc increases HA and glycosaminoglycan (GAG) synthesis of chondrocytes, exhibits a high degree of anti-inflammatory ability, and is capable of scavenging reactive oxygen species [18, 19]. Shikhman et al. proved that intramuscular injections of GlcNAc provide better chondroprotective and anti-inflammatory activity than HA in an anterior cruciate ligament transection model of OA using rabbits [13, 19]. In addition, our previous study demonstrated that intra-articular administration of GlcNAc promoted the repair of full-thickness articular cartilage defects in a rabbit knee joint model [20]. However, HA is another signal we used to compare osteochondral regeneration with GlcNAc. Intra-articular injections of HA (i.e., viscosupplementation) have been approved worldwide for OA treatment since the end of the 1980s. HA is responsible for the viscoelastic and rheological properties of synovial fluid and provides necessary lubrication, shock absorption, and friction reduction in joints [21]. Shim et al. found that the cell-rich supramolecular HA hydrogel with atelocollagen could allow outstanding regenerative ability for the reconstruction of osteochondral tissue in the knee joints of rabbits [22]. Furthermore, the Ica conjugated hyaluronic acid/collagen (Ica-HA/Col) composite hydrogels have been validated to facilitate the reconstruction of osteochondral interface in rabbit subchondral defects [23]. Such evidences suggested that HA possessed the potential to improve osteochondral regeneration.

In our previous study, intra-articular injection of GlcNAc could only promote the repair of experimental FTAC defects in the rabbit knee joint model [20]. However, lack of support from the scaffolds could cause subchondral bone overgrowth [24, 25]. In this study, we developed an intra-articular administration system of two different ECM materials (GlcNAc and HA) and combined it with cell-free porous PLGA graft implants using an in vivo animal model. Therefore, we aimed to investigate whether these two different ECM materials could lead to regenerative effects in osteochondral tissue, alone or in combination.

## Materials and methods

### Reagents

GlcNAc was obtained (SI-A3286; Sigma-Aldrich, St. Louis, MO), dissolved in normal saline, and filtered through 0.22-μm filters for sterilization. The sterilized solution was stored at 4°C. The HA used was Suplasyn (20 mg/2 mL; Bioniche, Galway, Ireland).

### Fabrication and characteristics of porous PLGA scaffolds

The porous PLGA (lactide/glycolide ratio, 85/15; molecular weight, 50–75 kDa; Sigma, St. Louis, MO) scaffold was fabricated using a salt-leaching technique with NaCl as the porogen. The entire procedure was the same as that performed during our previous study [26]. In brief, 5 mL of 20% (w/v) PLGA chloroform solution was mixed with 9 g of sodium chloride (NaCl) particles of 300 to 500 μm in diameter, yielding a 90% (w/v) solution that was cast into a multi-hole cylinder mold (3 mm in diameter and 3 mm in height) and lyophilized for 1 day to generate PLGA grafts. A scanning electron microscope (JEOL JSM-6700F) was used to observe the morphology of the pore structure, the interconnections, and the pore size of the PLGA scaffold. The average pore size was determined by using the Image J software program. The porous scaffold was sputter-coated with gold for 40 seconds at ambient temperature. Micrographs were obtained at 30× magnification with an accelerating voltage of 10 kV.

### Surgical procedure

All surgical procedures were approved by the Institutional Animal Care and Use Committee of National Cheng Kung University [20]. Male New Zealand White rabbits (Livestock Research Institute, Tainan, Taiwan) weighing 2.5–3 kg were used for all experiments. Before surgery, anesthesia was induced with Zoletil 50 (10–15 mg/kg; Virbac, Carros, France), and maintenance was achieved with a mixture of 2% isoflurane (Panion & BF Biotech Inc., Taipei, Taiwan) and oxygen/nitrous oxide (1/0.4 L/min).

Both knees of each rabbit were shaved and disinfected with a 10% ethanol-iodine solution. A 3-cm incision was made longitudinally along the parapatellar and capsular ligaments. The medial femoral condyle was exposed by lateral patellar dislocation. A full-thickness osteochondral defect 3 mm in diameter and 3 mm in depth was created with an electric drill on the medial femoral condyle and irrigated immediately with saline. PLGA scaffolds were pressed to fit into the defects.

Postoperatively, the rabbits were housed in a standardized cage under a 12 hour light–dark cycle and allowed food and water ad libitum. An antibiotic (25 mg/kg, Enrofloxacin) and analgesic (Ketoprofene) were administered to the rabbits for 3 days. Diarrhea, appetite, body weight, and wound healing were monitored during experiment. The remaining animals were euthanaized with overdose anesthetic and intravenous injection of potassium chloride (1-2meq/kg) after four or twelve weeks.

### Intra-articular injections

**Fig 1:**
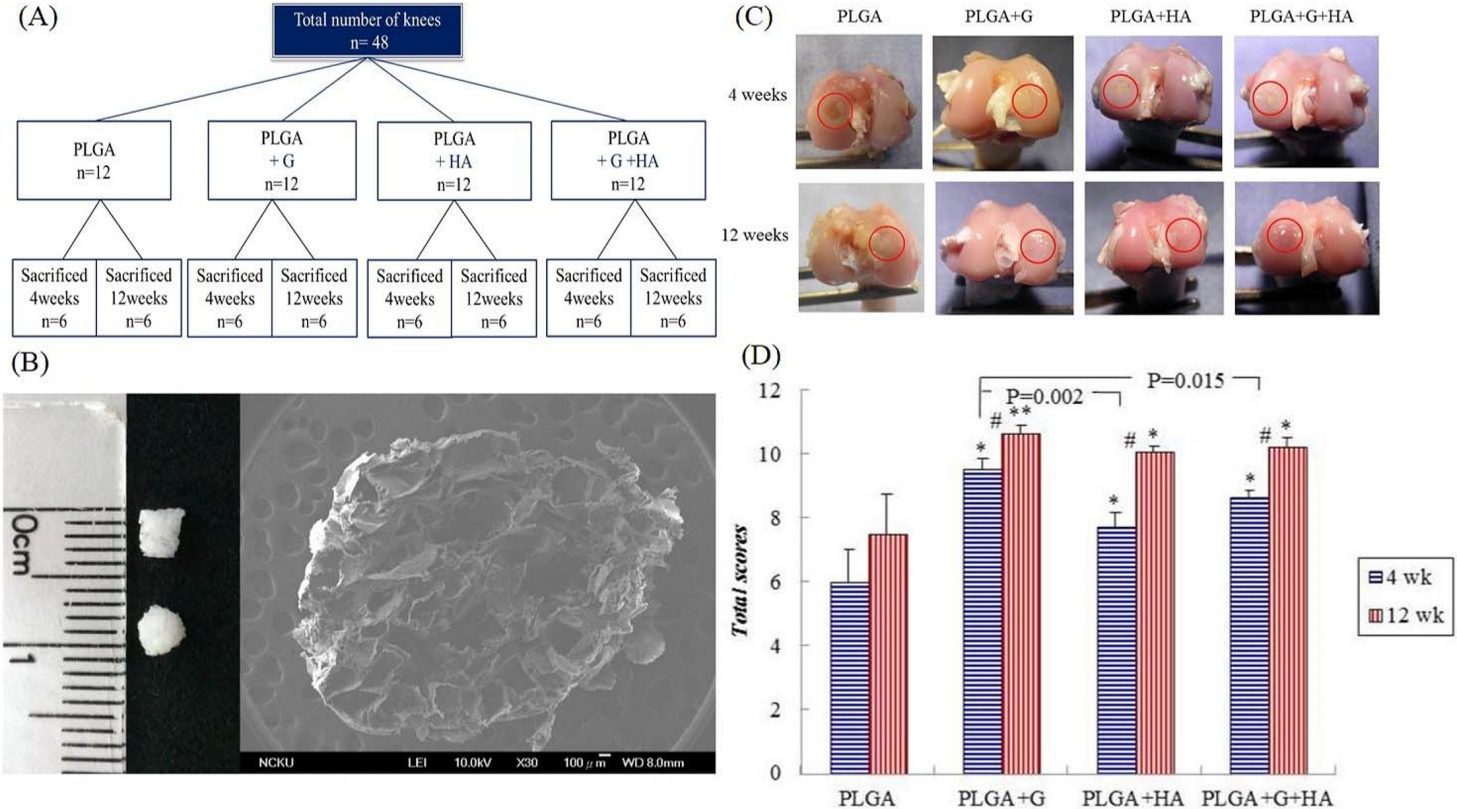
(A) Schematic diagram of the animal study groups. (B) PLGA sponge scaffold. Photograph image shows the dimension of the PLGA scaffolds. Scanning electron microscope image shows the porous structure of the PLGA scaffold. (C) Representative images of the gross appearances of the four groups at 4 weeks and 12 weeks after surgery. Circles surround the repaired osteochondral defect area. (D) Quantitative scores of the gross appearances of the four groups at 4 weeks and 12 weeks. #Between two time points, p<0.05. *Compared with the PLGA group, p<0.05. **Compared with the PLGA group, p<0.001.

All rabbits were randomly divided into four groups. No injection was used for the PLGA group. The PLGA+HA and PLGA+GlcNAc (G)+HA groups were administered intra-articular injections of HA and HA/GlcNAc, respectively, once per week starting 1 week postoperatively for 3 weeks. The PLGA+G group was administered intra-articular injections of GlcNAc twice per week starting 1 week postoperatively for a period of 4 weeks. Six rabbits in each group were sacrificed at 4 weeks and 12 weeks after surgery (Fig 1A). The dose of GlcNAc was 80 mg/0.3 mL per joint. The HA+G solution was GlcNAc directly dissolved in HA.

### Macroscopic evaluations

After sacrifice at postoperative week 4 or week 12, the macroscopic and histological scores of the regenerated tissue were blindly assessed by two investigators according to the modified Wayne’s grading scale (S1 Table) [27]. The score of each group was calculated and statistically analyzed. The digital femoral condyle was fixed in 10% neutral buffered formaldehyde water solution until preparation for micro-computed tomography (micro-CT) and histological examination.

### Micro-CT analysis

All samples were scanned using a high-resolution microtomograph 1076 scanner (Skyscan, Kontich, Belgium) to obtain both qualitative and quantitative measurements of the bone regeneration level within the defects. A 360-degree scan was performed at a voltage of 50 kV and a current of 160 μA. Three-dimensional reconstructions were created using the Skyscan CT-Analyser program (version 1.8, data software), and a cylindrical region of interest with a diameter of 3 mm (diameter of defect) was used for analysis. The bone volume per tissue volume (BV/TV) and trabecular thickness (Tb.Th) were obtained to evaluate the bone volume density and to measure the thickness of trabecular structures, respectively.

### Histology

All histological sections were performed by the Department of Pathology at Chi-Mei Medical Center in Tainan. The femur ends were harvested, fixed in 10% neutral buffered formaldehyde solution, decalcified, and embedded in paraffin blocks. For each sample, the tissue was sliced using a microtome into 4-μm-thick sections for different histological staining procedures. Hematoxylin and eosin staining was used for general observations, and Masson’s trichrome stain was used for total collagen and alignment. Alcian blue stain was used for GAG synthesis staining. The sections were observed by light microscopy (Olympus IX71; Olympus Tokyo, Japan) and documented using a digital charge-coupled device camera (Olympus DP72; Olympus).

Histology sections were evaluated using a modified O’Driscoll system [28]. This well-recognized grading scheme was blindly assessed by two investigators, and the maximum total score was 34 points (Table 1). The total score of each group was calculated and statistically analyzed.

**Table 1:** Modified O’Driscoll histological score for repair evaluation.

**a. Overall defect evaluation (include entire defect)**		**Points**
1. Percent filling with neo-formed tissue	75–100%	3
	50–75%	2
	25–50%	1
	0–25%	0
**b. Subchondral bone evaluation**		**Points**
2. Percent filling with neo-formed tissue in subchondral bone	75–100%	3
	50–75%	2
	25–50%	1
	0–25%	0
3. Subchondral bone morphology	Normal, trabecular bone	4
	Trabecular, with some compact bone	3
	Compact bone	2
	Compact bone and fibrous tissue	1
	Only fibrous tissue or no tissue	0
4. Extent of neo-tissue bonding with adjacent bone in the subchondral bone	Complete on both edges	3
	Complete on one edge	2
	Partial on both edges	1
	Without continuity on either edge	0
**c. Cartilage evaluation**		**Points**
5. Morphology of neo-formed surface tissue	Mainly hyaline cartilage	6
	Fibrocartilage (spherical cell morphology >75% of cells)	4
	Only fibrous tissue (spherical cell morphology <75% of cells)	2
	No tissue	0
6. Thickness of neo-formed cartilage	Similar to the surrounding cartilage	3
	Greater than surrounding cartilage	2
	Less than the surrounding cartilage	1
	No cartilage	0
7. Joint surface regularity	Smooth, intact surface	3
	Surface fissures (<25% neo-surface thickness)	2
	Deep fissures (25–99% neo-surface thickness)	1
	Complete disruption of the neo-surface	0
8. Chondrocyte clustering	None	3
	Less than 25% of chondrocytes	2
	25–100% chondrocytes	1
	No chondrocytes present (no cartilage)	0
9. Chondrocyte and GAG content of neo-cartilage	Normal cellularity with normal Alcian blue staining	3
	Normal cellularity with moderate Alcian blue staining	2
	Obviously fewer cells with poor Alcian blue staining	1
	Few cells with no or little Alcian blue staining or no cartilage	0
10. Chondrocyte and GAG content of adjacent cartilage	Normal cellularity with normal Alcian blue staining	3
	Normal cellularity with moderate Alcian blue staining	2
	Obviously fewer cells with poor Alcian blue staining	1
	Few cells with no or little Alcian blue staining or no cartilage	0
11. Cartilage vascularization	Absent	1
	Present	0
***Maximum possible total score***		**34**
Percent degradation of the PLGA (if any)	75–100%	3
	50–75%	2
	25–50%	1
	0–25%	0

### Immunohistochemistry

Immunohistochemistry was performed to detect the expression of collagen type I (COL I; fibrocartilage), collagen type II (COL II; hyaline cartilage), matrix metalloproteinase (MMP)-13, endogenous growth factors (i.e., transforming growth factor [TGF]-β2 and TGF-β3), and Sox9 (marker of mature chondrocytes) for osteochondral defect regeneration.

After rehydration, H_2_O_2_ was used to block the endogenous peroxidase. The specimens were incubated with proteinase K intended for proteolytic digestion of the formalin-fixed protein cross-link and then treated with immunoblotting solution. The specimens were incubated overnight with a monoclonal primary antibody diluted 1:100 (COL I, Bioworld Technology; COL II, Bioss; TGFβ-2 or TGFβ-3, Spring Bioscience; SOX9, Bioss; or MMP-13, Bioworld Technology) and the second antibody (DAB [3,3ʹdiaminobenzidine]). Hematoxylin staining was performed (rabbit/mouse HRP-DAB polymer detection system, Cat. No. BSB0205; BioSB, CA, USA), and DAB presented a brown color, indicating immunopositivity.

### Statistical analysis

SPSS version 17.0 was used for data analysis. All data are expressed as mean±standard error of the mean. Data were not normally distributed; therefore, the homogeneity of variance was corroborated by Levene’s test. Comparisons among the groups at each time point were analyzed using the Mann-Whitney U-test with the nonparametric Kruskal-Wallis post-test; p<0.05 were considered statistically significant.

## Results

### Characteristics of the three-dimensional PLGA sponges

The porous PLGA scaffolds used in this study were 3 mm in diameter and 3 mm in height, and the inner porous structure was observed using a scanning electron microscope (Fig 1B). The average diameters of the pores ranged from 300 to 500μm, and the PLGA scaffold with a porosity of over 90% showed an interconnected open-pore structure. Our previous study showed that PLGA scaffolds have suitable mechanical strength and degradation rates. The compressive modulus of the PLGA scaffolds was 0.65±0.11 MPa in the wet state, which was similar to normal cartilage in rabbits (0.55±0.01 MPa) [29].

### Macroscopic evaluations and quantitative scores

#### Macroscopic evaluations at 4 weeks

At 4 weeks postoperatively, all defect areas were completely filled with repaired tissue except for those in the PLGA-only group (Fig 1C). In the PLGA group, the area showed mild depressions in the defect site and the rim was surrounded with white regenerative tissue for ongoing repair. Regarding the PLGA+G, PLGA+HA, and PLGA+G+HA treatment groups, the partially degraded PLGA scaffold remained visible in the center of the defect. However, the surrounding cartilage was the same color as the normal cartilage, thus representing ongoing repair of the hyaline-like cartilage.

#### Macroscopic evaluations at 12 weeks

At 12 weeks postoperatively, the defect areas in all groups were completely filled with repaired tissue (Fig 1C). In the PLGA group, the defects were apparently covered with opaque tissue. In contrast, in the PLGA+G, PLGA+HA, and PLGA+G+HA groups, the repair cartilage became more mature, had the appearance of a smooth joint surface, and the color closely resembled that of the adjacent normal hyaline cartilage.

#### Quantitative scores of gross appearances

The gross appearances according to the modified Wayne’s grading scale scoring system (S1 Table.) of the four groups are shown (Fig 1C). At 4 weeks, the scores of the PLGA+G, PLGA+HA, and PLGA+G+HA groups were significantly higher than those of the PLGA group, especially the PLGA+G (p<0.001) group. The score of the PLGA+G group was significantly higher than that of the PLGA+HA group (p = 0.002) and PLGA+G+HA group (p = 0.015). However, there was no statistically significant difference between the PLGA+HA group and PLGA+G+HA group.

Similar to the results at 4 weeks, the scores of the PLGA+G, PLGA+HA, and PLGA+G+HA groups were significantly higher than those of the PLGA group at 12 weeks. Moreover, there were no statistically significant difference among the PLGA+G, PLGA+HA, and PLGA+G+HA groups at 12 weeks. In addition, the PLGA+G (p<0.001), PLGA+HA (p<0.001), and PLGA+G+HA (p = 0.001) groups exhibited significant differences between 4 and 12 weeks.

### Micro-CT analysis

#### Micro-CT image analysis

**Fig 2:**
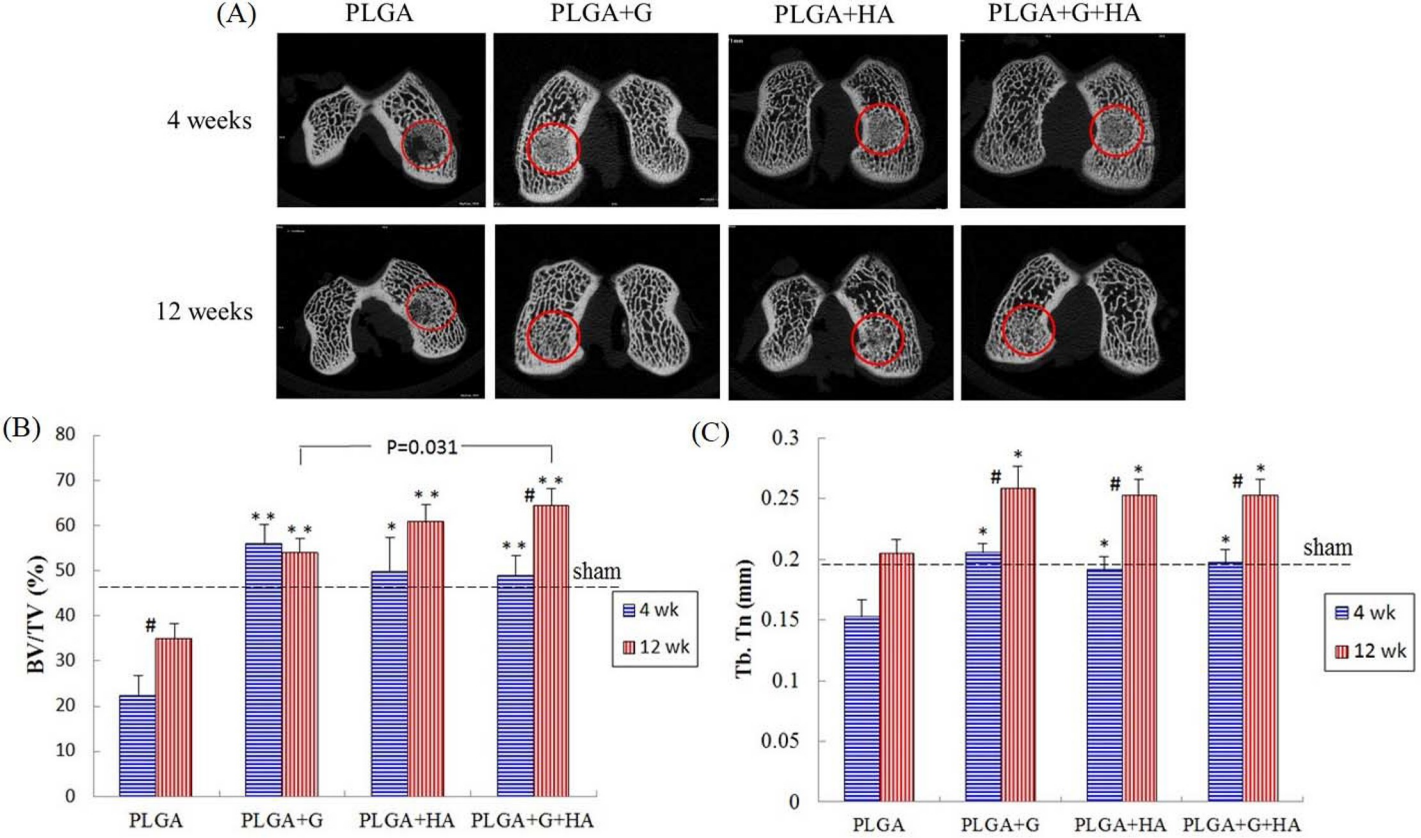
(A) The micro-CT images for bone assessment of each group at 4 weeks and 12 weeks after surgery. Circles surround the repaired osteochondral defect area. (B) Quantification scores of BV/TV. (C) Quantification scores of the Tb.Th. #Between two time points, p<0.05. *Compared with the PLGA group, p<0.05, **Compared with the PLGA group, p<0.001.

Horizontal micro-CT images of the medial condyles of each group at 4 weeks and 12 weeks postoperatively are shown (Fig 2A). More newly mineralized tissue was observed in the PLGA+G, PLGA+HA, and PLGA+G+HA groups than in the PLGA-only group at 4 weeks and 12 weeks. In the PLGA+G and PLGA+G+HA groups, the defect areas were similar to normal bone structure at 12 weeks.

#### Quantitative scores of micro-CT images

At 4 weeks, the BV/TV ratios of the PLGA+G (55.89±4.34), PLGA+HA (49.74±7.54), and PLGA+G+HA (48.84±4.47) groups were significantly higher than those of the PLGA (22.25±4.5) group (p<0.001, p = 0.004, and p<0.001, respectively) (Fig 2B). The Tb.Th values of the PLGA+G (0.21±0.01), PLGA+HA (0.19±0.01), and PLGA+G+HA (0.20±0.01) groups were significantly higher than those of the PLGA (0.15±0.01) group (p<0.001, p = 0.02, and p = 0.003, respectively) (Fig 2C). The BV/TV ratio and Tb.Th values of the intra-articular injected groups were not significantly different compared with those of the sham group (Fig 2B and 2C).

At 12 weeks, the BV/TV ratios of the PLGA+G (53.96±3.22), PLGA+HA (60.72±3.9), and PLGA+G+HA (64.47648.84±3.64) groups were significantly higher than those of the PLGA (34.93±3.33) group (p<0.001, p<0.001, and p<0.001, respectively). In addition, the BV/TV ratio of the PLGA+G+HA (p = 0.031) group was significantly higher than that of the PLGA+G group at 12 weeks (Fig 2B). The Tb.Th values of the PLGA+G (0.26±0.02), PLGA+HA (0.25±0.01), and PLGA+G+HA (0.25±0.01) groups were significantly higher than those of the PLGA (0.2±0.01) group (p = 0.001, p = 0.01, and p = 0.007, respectively) (Fig 2C). However, the Tb.Th values of the intra-articular injection groups were similar to those of the sham group, and there was no significant difference among them at 12 weeks (Fig 2C).

The PLGA (p = 0.04) and PLGA+G+HA (p = 0.002) groups exhibited significant differences in BV/TV ratios between 4 weeks and 12 weeks. All of the groups (p = 0.004, p<0.001, p = 0.001, and p = 0.004, respectively) exhibited significant differences in Tb.Th values between 4 weeks and 12 weeks.

### Histological and immunohistochemical analysis

#### Histological examination

**Fig 3:**
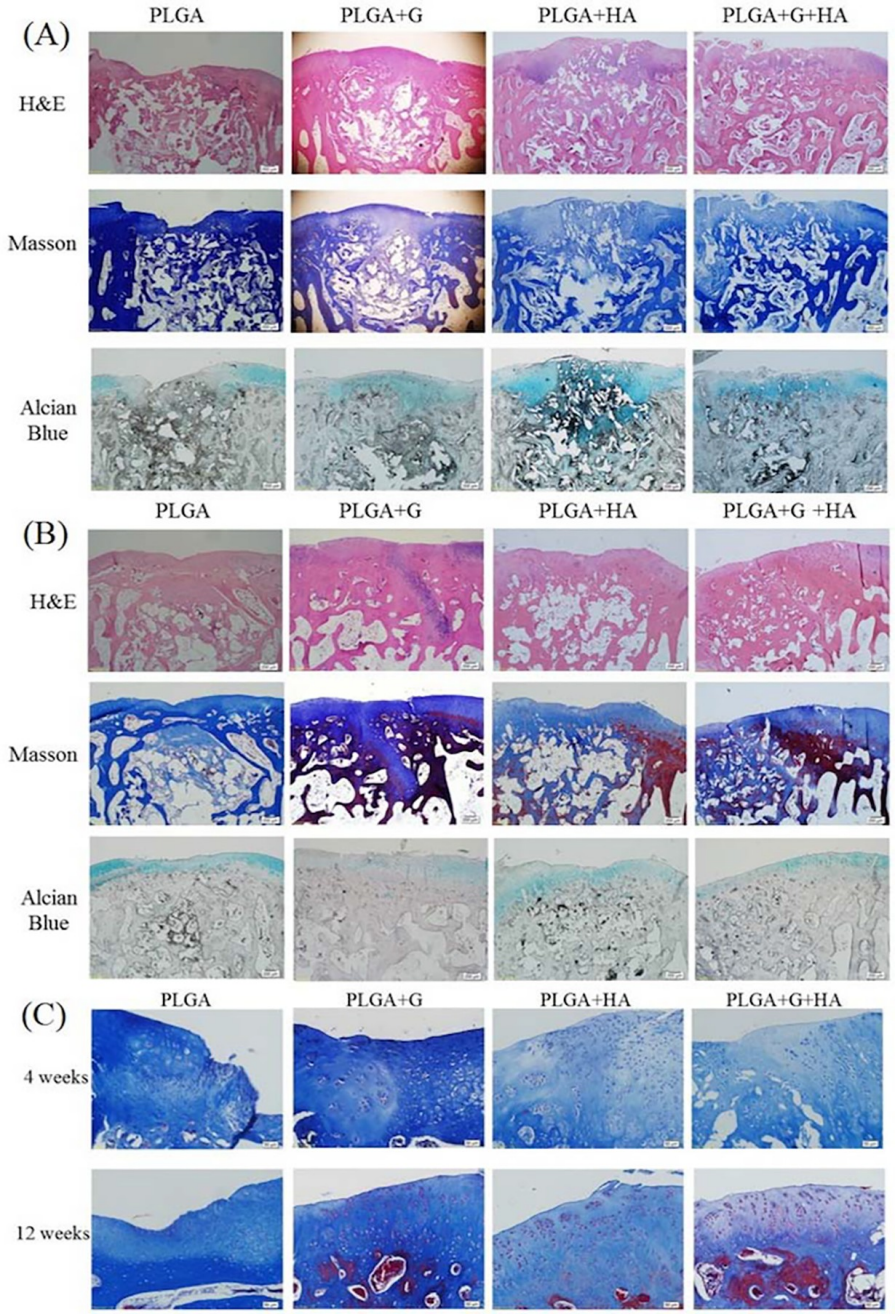
Representative images of (A) Histological examinations with staining of the repaired area using hematoxylin and eosin (H&E), Masson’s trichrome, and Alcian blue at 4 weeks. Scale bar: 200 μm. (B) Histological examinations with staining of the repaired area using H&E, Masson’s trichrome, and Alcian blue at 12 weeks. Scale bar: 200 μm. The region of yellow arrows indicated the original outline of the implant. (C) Higher magnifications of the defect areas of each group using Masson’s stain. Scale bar: 50 μm.

At 4 weeks, the surface of the PLGA scaffold had degraded but partially remained in the defect site. The new cartilage and bone tissue grew and penetrated and replaced the scaffold gradually. In the PLGA group, disrupted and depressed surfaces were observed, and the defect sites were filled with fibrous or immature repaired tissue (Fig 3A). Furthermore, GAG expression only occurred in the adjacent host, and not on the surface of the defect. With degradation of the PLGA scaffold, there was less new tissue ingrowth into the defect sites and fewer regenerative tissues and junctions between the repaired tissue and surrounding native tissue.

However, in the PLGA+G, PLGA+HA, and PLGA+G+HA groups, the surface of the defects appeared smoother and had more GAG expression in the repaired cartilage (Fig 3A), and the defect adjacent to the host was mostly covered with both hyaline and fibrous cartilage. Additionally, most of the repaired cartilage obviously consisted of ovoid cells in the lacunae (Fig 3C).

At 12 weeks, the PLGA scaffolds were almost degraded and the repaired tissue became more mature. In the PLGA group, the regenerated cartilage was mostly fibrous and less hyaline-like. In some samples of the PLGA+G group, even though the cartilage surface was smoother and the thickness made it closer to the host, there was less GAG content and gap formation in the subchondral site. In contrast, there was no gap formation at the subchondral site, rich GAG content, and an interrupted surface in the PLGA+HA group. However, the PLGA+G+HA group demonstrated essentially original hyaline cartilage structures that appeared to have sound chondrocyte orientation, a considerable level of GAG, and reconstruction of the bone structure (Fig 3B).

#### Total histological modified scale scores

**Comparisons of specific parameters between the four groups of histological scale**

At 4 weeks, the total scores of the PLGA+G, PLGA+HA, and PLGA +G+HA groups were significantly higher than those of the PLGA group (p = 0.001, p = 0.003, p<0.001) (Table 2). However, the PLGA+G+HA group exhibited significant bone reconstruction (e.g., bone filling, subchondral morphology), chondrocyte clustering, and interaction with adjacent surfaces in the four groups. In particular, degradation of the PLGA scaffolds in combination with GlcNAc and HA was more than 75%.

**Table 2:** Total histological modified scale scores.

	4 weeks				12 weeks			
	PLGA	PLGA+G	PLGA+HA	PLGA+G+HA	PLGA	PLGA+G	PLGA+HA	PLGA+G+HA
Overall filling	0.83±0.6	1.7±0.3	1.9±0.1	2.5±0.3^a^	1.8±0.4	2.5±0.3	2.3±0.3	2.7±0.3
Bone filling	0.25±0.25	1.1±0.1^a^	1.6±0.3^a^	2.8±0.3^b^^d^^e^	1.3±0.3	2.2±0.4	2.3±0.3^a^	2.9±0.1^b^
Subchondral morphology	0.5±0.3	0.6±0.3	1.3±0.3	2.4±0.2^b^^d^^e^	1.3±0.3	2.1±0.5^#^	2.9±0.3^a^^#^	3.2±0.2^b^^#^
Bone bonding	0.4±0.2	1.2±0.4	1.7±0.7	2.6±0.6^a^	1.9±0.6	2.1±0.7	2.5±0.7	3.3±0.4
Surface morphology	1.3±0.7	3.3±1.2	3.5±0.3^a^	4.3±0.9^a^	3.0±0.6	3.8±0.4	4.2±0.7	5.2±1.1
Cartilage thickness	0.17±0.2	2.4±0.8	2.5±0.8^a^	2.4±0.9	2.2±1.2	2.1±1.2	3.2±1.5	3.1±1.5
Surface regularity	0.7±0.3	1.8±0.6	1.2±0.2	2.1±0.6	1.7±0.3	1.4±0.3	2±0.6	2.6±0.3^c^
Chondrocyte clustering	0.1±0.1	2.0±0.6^a^	1.0±0.0^b^	1.8±0.2^b^^e^	0.9±0.5	1.8±0.4	1.9±0.1*	2.8±0.3^a^^c^^e^^#^
GAG and cell content of neo-surface	0.2±0.2	1.5±0.3^a^	1.3±0.3^a^	1.8±0.2^b^	1.1±0.1	1.5±0.3^#^	1.9±0.1^b^	2.3±0.3^a^^c^
GAG and cell content of adjacent surface	0.5±0.3	1.3±0.4	1.8±0.3^a^	1.8±0.2^a^^d^	1.8±0.3	1.3±0.3^#^	1.7±0.4	2.8±0.3^a^^d^^e^^#^
Total PLGA degradation	4.9±1.3 0.2±0.1	16.8±1.1^b^ 0.7±0.3	17.7±1.8^b^ 1.2±0.2^a^	24.5±1.5^b^^e^ 3.0±0.0^b^	16.9±2.9^#^ 0.8±0.2^d^	21.7±2.4^#^ 2.5±0.3^b^^d^*	24.8±1.9^a^^#^ 2.2±0.1b^#^	30.1±1.2^a^^c^^e^^#^ 3.0±0.0^b^

Values were expressed as mean±standard error of the mean for each parameter

^a^Compared with the PLGA group at the same time point (p<0.05).

^b^Compared with the PLGA group at the same time point (p<0.01).

^c^Compared with the PLGA+G group at the same time point (p<0.05).

^d^Compared with the PLGA+G group at the same time point (p<0.01).

^e^Compared with the PLGA+HA group at the same time point (p < 0.05).

^f^Compared with the PLGA+HA group at the same time point (p<0.01).

^#^Compared to 4 weeks postoperatively (p<0.05).

*Compared to 4 weeks postoperatively (p<0.01).

Regarding the total scores at 12 weeks, those of the PLGA+HA (p = 0.048) and PLGA+G+HA (p = 0.014) groups were significantly different from those of the PLGA group, whereas the PLGA+G (p = 0.14) group did not have a significantly different total score. However, the total score of the PLGA+HA group (24.8±1.9) was higher than that of the PLGA+G group (21.7±2.4), without significant difference. Moreover, the total score of the PLGA+G+HA group was significantly different from that of the PLGA (p = 0.014), PLGA+G (p = 0.04), and PLGA+HA (p = 0.035) groups. Histological assessments of the PLGA+G+HA group to compare specific parameters revealed the best chondrocyte clustering and GAG and cell content.

**Comparisons of results at 4 weeks and 12 weeks using the histological scal**

All groups had significant differences in total histological scores (PLGA, p = 0.019; PLGA+HA, p = 0.026; PLGA+G+HA, p = 0.045), except for the PLGA+G group (p = 0.08) (Table 2). At 12 weeks, the PLGA+G group had significantly better subchondral morphology (p = 0.03) and GAG and cell content (surface of neo-tissues, p<0.01; adjacent surface, p = 0.015), and the PLGA+HA group had significantly better subchondral morphology (p = 0.012) and chondrocyte clustering (p<0.01) compared to the values at 4 weeks. Notably, at 12 weeks, the PLGA+G+HA group exhibited effects similar to those of the PLGA+G and PLGA+HA groups, of which subchondral morphology (p = 0.02), chondrocyte clustering (p = 0.02), and GAG and cell content of the adjacent surface (p = 0.02) were significantly higher than those at 4 weeks.

**Immunohistochemical examination**

**Fig 4:**
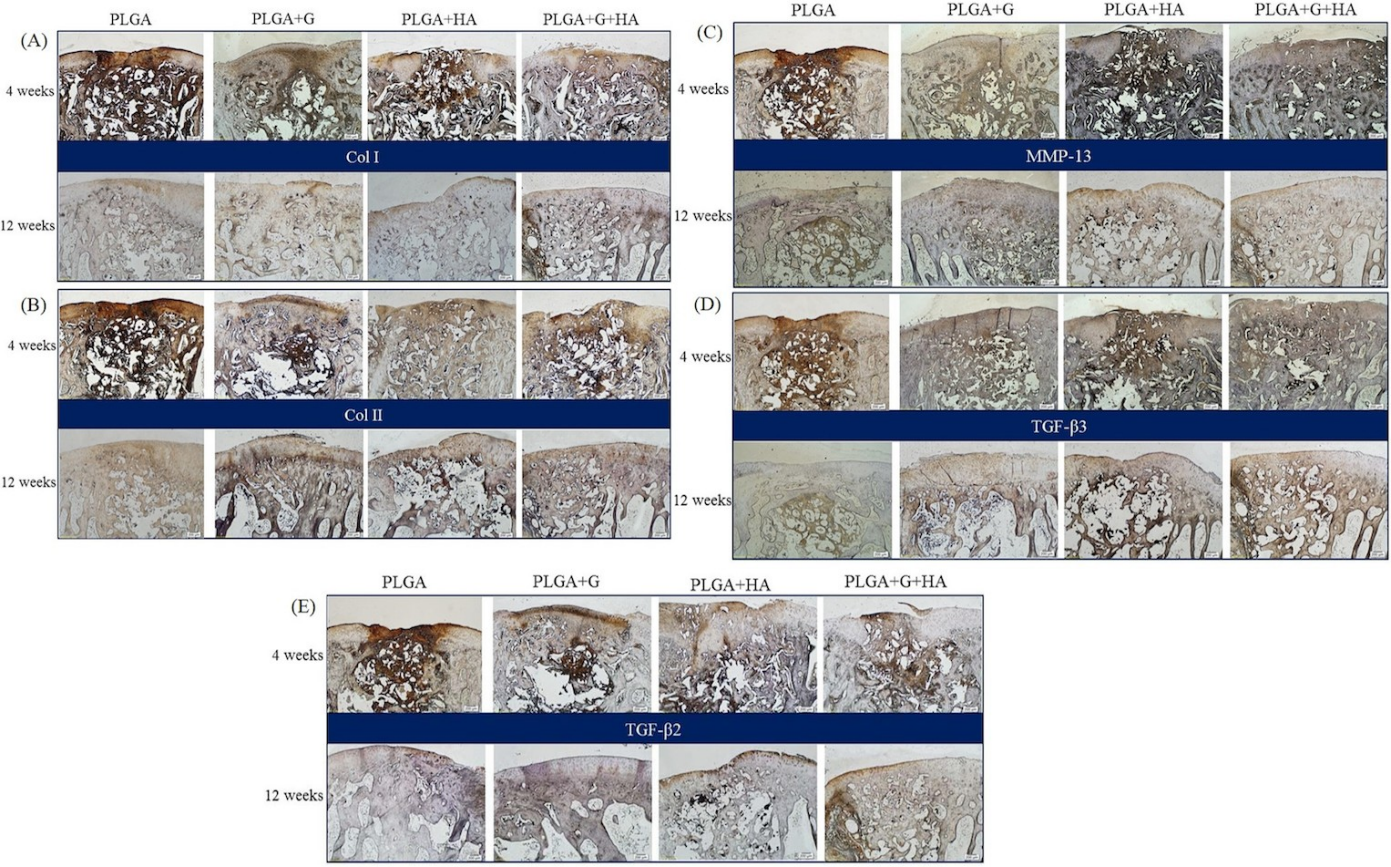
Representative images of immunohistochemistry in each group. (A) Collagen type I. (B) Collagen type II. (C) MMP-13. (D) TGF-β3. (E) TGF-β2. The region of yellow arrows indicated the original outline of the implant. Scale bar: 200 μm at 4 weeks and 12 weeks.

Expressions of COL I and COL II were strong at the defect sites at 4 weeks in all groups (Fig 4A and 4B). However, at 12 weeks, the repairing defect area in the PLGA group had fibrous tissues at the surface that contained mostly COL I and less COL II. In contrast, the repairing defects in the PLGA+G, PLGA+HA, and PLGA+G+HA groups had abundant COL II expression, which was an important indicator of hyaline cartilage formation (Fig 4B) and positive Sox9 expression corresponding with chondrocytes in the lacunae (S1 Fig.).

### Discussion

Early intervention with scaffolds with comparable stiffness created better conditions for subchondral bone formation and subsequently enhanced cartilage regeneration [30]. After autologous chondrocyte implantation surgery, 25% and 34% of patients were found to have upward migration of subchondral bone [31, 32], especially in the weight-bearing regions [31]. The PLGA scaffolds that we used in our study provided a supportive matrix and encouraged the ingrowth of cells and tissues. The PLGA scaffold remnants were still visible and gradually replaced with reconstituted tissue at 4 weeks. After 12 weeks, the implants were almost completely degraded and replaced with regenerated tissue in the defect sites in all groups, especially the PLGA+G+HA group (Fig 3B). This is consistent with a previous study [33], in which the degradation half-time of the PLGA scaffold was found to be 3–4 weeks in vitro. With regard to the inflammation response of the PLGA scaffold, representative cells such as plasma cells, lymphocytes, and multinucleated giant cells in subchondral reparative tissues were evident at 6 weeks and gradually decreased at 12 weeks [34]. However, with the mechanical support of the PLGA scaffold, intra-articular injections of GlcNAc could significantly improve the degree of osteochondral regeneration over that of the comparative data (S2 and S3 Figs) from the previous study [20].

The microenvironment plays a critical role in determining the repair of articular cartilage. Intra-articular injections of GlcNAc with acellular PLGA scaffolds could directly meet the in-situ requirement of microenvironment growth rather than being substantially absorbed by the gastrointestinal tract through oral administration. Compared with the PLGA scaffold-only group, the PLGA+G group exhibited smooth cartilage (Fig 1C and 1D) and displayed hyaline-like cartilage regeneration, GAG content, positive COL II content (Figs 3 and 4), and significantly calcified tissue (BV/TV and Tb.Th) (Fig 2). In our previous study, without the mechanical support of the scaffold, the surfaces of the regenerated areas were still concave at 4 weeks [20]. In addition, with the support of the PLGA scaffold, the concave area of the defect could be filled at an early stage, and the administration of GlcNAc not only improved extracellular matrix deposition in the cartilage but also promoted bone regeneration. GlcNAc and GlcN have been shown to increase bone mineralization, stimulate osteoblastic differentiation, and inhibit osteoclast differentiation and activation [35].

The biological effects of HA depend on its molecular weight [36–38]. In the present study, Suplasyn (Bioniche), the molecular weight of which is approximately 500–1,000 kDa, comprises high-molecular-weight HA. High-molecular-weight HA has viscoelastic properties similar to those of synovial fluid and has anti-angiogenic [39], anti-inflammatory [40], and chondroprotective effects that reduce pain and disability and improve joint function [41]. However, some degradation products of HA called oligosaccharides of HA were shown to stimulate vascular endothelial cell proliferation in specific sizes (3–10 disaccharide units) [42–46] and pro-angiogenic factors such as vascular endothelial growth factor, which is an essential mediator of both angiogenesis and endochondral ossification [47]. Fortunately, other non-degradation high-molecular-weight HA might inhibit this situation [43] and prevent overgrowth of bone formation. In the present study, the PLGA+HA group exhibited hyaline cartilage formation near the host site, and bone tissue was gradually replaced by the degraded part of the PLGA scaffold at 4 weeks. Moreover, COL II-based neo-tissues were well-integrated in the adjacent surrounding cartilage and were anchored in situ on the newly generated lamellar bones at 12 weeks.

Regarding the PLGA+G+HA groups, when the PLGA scaffolds gradually degraded, the defects were filled with newly formed matrix tissue at appropriately 4 weeks, especially in the subchondral bone (Fig 3). Some reports have demonstrated that small nutrients and signal molecules might diffuse from the subchondral to deep cartilage [48, 49], and this should be the second route of the exchanged transport of nutrients and oxygen in articular cartilage. However, the primary exchange transport relies on the synovial membrane and synovial fluid. When GlcNAc and HA were administered as combination treatment, it seemed that the regeneration stage could be triggered earlier than when administered alone. Eventually, organized collagen deposition, predominantly COL II, greater GAG synthesis in the regenerating cartilage, and sound subchondral bone formation were established in the PLGA+G+HA group (Figs 3 and 4). The possible mechanism might have been the early subchondral bone regeneration and continuous administration of HA and GlcN; these two key components of the extracellular matrices in normal articular cartilage would provide a favorable environment for mesenchymal stem cell chondrogenesis [50, 51].

In this study, we used intra-articular injections for the rabbit osteochondral model. Due to the short half-life of HA, which is 0.5 to 1 days in rabbits [52], hyaluronidase degrades HA into HA fragments or oligosaccharides of HA, which might cause bone formation. The solution might be used with much higher-molecular-weight HA such as Synvisc (molecular weight, 6000 kDa) or chemically cross-linked HA-encapsulated GlcNAc for controlled release. However, we think that intra-articular injections every week might produce greater improvements in quality of life for longer periods.

The TGF-β superfamily plays important roles in promoting chondrocyte growth, proliferation, and differentiation (including osteochondrogenic differentiation) [53]. TGF-β3 chemotactically recruited stem cells and induced chondrogenesis of the recruited cells in vitro [54]. In addition, TGF-β2 stimulated chondrocyte proliferation [55]. However, with the administration of HA and GlcNAc alone or together, the TGF-β superfamily was exhibited in defect areas. Most importantly, PLGA+G+HA expressed considerable tissue levels of TGF-β2 and TGF-β3 in the repaired sites at 4 weeks that were sustained until 12 weeks compared with other groups (Fig 4D and 4E). We suggested that the diffusion of nutrients, growth factors, and cytokines might accompany subchondral bone regeneration to provide a suitable microenvironment for osteochondral repair.

MMP-13 plays a significant role in the catabolism of articular cartilage [56]. At an early time point, high levels of MMP-13 expression were exhibited by all groups, but they later disappeared with bone remodeling (Fig 4C). SOX9 is a transcription factor for COL II expression in prehypertrophic chondrocytes [57]. In our study, positive expression of SOX9 in the PLGA+G, PLGA+HA, and PLGA+G+HA groups was observed at 4 weeks and 12 weeks (S1 Fig). Both GlcNAc and HA can potentially induce chondroprotection and inflammation inhibition in cartilage regeneration.

In our study, we used a monophasic, cell-free, and synthetic PLGA scaffold for osteochondral regeneration. Despite the lack of physical or biochemical cues that multiphasic scaffolds can provide for specific targeting of cartilage or bone development [58, 59], precautions over scaffold separation in vivo are unnecessary. With regard to the administrated supplements, Shikhman et al. found that the chondroprotective efficacy of GlcNAc was better than that of viscosupplementation treatment with HA in the OA model [13]. In our study, there were no significant differences between the PLGA+G and PLGA+HA groups. The reason might be the high molecular weight of HA, and the different severities of articular cartilage lesions in the present study. Shim et al. have validated the potential of an HA and atelocollagen combination for osteochondral regeneration [22]. This effective ability of HA to mediate osteochondral tissue regeneration has also been confirmed in the present study.

There are still some limitations in this study. First, the injection period was different in this two supplements. The PLGA+HA and PLGA+G+HA groups were administered injections once per week for 3 weeks based on the intra-articular injection of HA for clinical OA treatment, but the PLGA+G group administered injections twice per week for 5 weeks was according to previous study [20]. The injection intervals of these supplements should be modified to be identical, and their consequences on osteochondral repair should be evaluated. Understanding the effects of these intervals on osteochondral repair will offer great clinical advantages for patients in the future. With the combination of HA, the injection interval of GlcNAc could be prolonged to once per week for 3 weeks and the results showed outstanding regenerative performance for the reconstruction of an osteochondral tissue in the knee joints of rabbits.

Second, we did not investigate the remnants of GlcNAc and HA in synovial fluid after 4 and 12 weeks, because it was difficult to harvest the pure synovial fluid (without dilution and bloodless) from rabbits. GlcNAc and HA are the main extracellular matrix in the cartilage, it is hard to distinguish whether they already exist in host or from intra-articular injection. Future studies should label the supplements before intra-articular injection and analyze the molecular weight of remnants by gel permeation chromatography in order to confirm the degradation rate in articular cartilage. Therefore, evidences revealed that this strategy opens an avenue for the combination of GlcNAc, HA and PLGA scaffolds with synergistic effects to obtain even greater therapeutic efficacy in osteochondral regeneration.

## Conclusions

In summary, we demonstrated that acellular PLGA scaffolds combined with intra-articular injections of GlcNAc and HA could accelerate osteochondral regeneration in rabbits. The three-dimensional acellular and porous PLGA scaffolds enhanced osteochondral regeneration and provided the rigidity to withstand the strength of compression loading at the weight-bearing site.

Interestingly, both intra-articular injections of HA and oral intake of glucosamine were originally used for OA treatment. In our study, intra-articular injection of these two supplements combined with acellular PLGA scaffolds created synergistic effects for osteochondral regeneration without the supplement of exogenous growth factors. Cartilage regeneration with great gross appearance, hyaline-like cartilage, bone regeneration, good collagen alignment, and abundant GAG expression occurred. As opposed to cell-based therapies, this implant combined with an intra-articular injection system would be a simple, effective, and clinically feasible therapeutic method for osteochondral regeneration.

## Supporting information

S1 Fig Immunohistochemistry images of the defect areas of Sox9. Scale bar: 50 ‘m at 4 weeks and 12 weeks.
(TIFF)

S2 Fig **The images of gross appearances in G and PLGA+G groups (A) and Quantitative scores (B) at 4 weeks and 12 weeks after operation.** Circles enclose the repaired osteochondral defect area. #: between two time point, *p*<0.05.
(TIF)

S3 Fig **The micro-CT images of bone assessment in G and PLGA+G groups (A) and quantification scores of the TV/BV (B) and Tb.Th (C) at 4 weeks and 12 weeks after operation.** Circles enclose the repaired osteochondral defect area. #: between two time point, *p*<0.05.
(TIFF)

S1 Table A modified Wayne’s grading scale scoring system for gross appearance. (DOCX)

## References

[1] Shea KG JJJ, Carey JL, Anderson AF, Oxford JT. Osteochondritis Dissecans Knee Histology Studies Have Variable Findings and Theories of Etiology. Clin Orthop Relat Res. 2012. 10.1007/s11999-012-2619-6 PMC3586021 23054514

[2] Yang KGA, Saris DBF, Geuze RE, Van der Helm YJM, Van Rijen MHP, Verbout AJ, et al Impact of expansion and redifferentiation conditions on chondrogenic capacity of cultured chondrocytes. Tissue Eng. 2006;12(9):2435–47. 10.1089/ten.2006.12.2435 16995777

[3] Martin I, Miot S, Barbero A, Jakob M, Wendt D. Osteochondral tissue engineering. J Biomech. 2007;40(4):750–65. 10.1016/j.jbiomech.2006.03.008 16730354

[4] Mano JF, Reis RL. Osteochondral defects: present situation and tissue engineering approaches. Journal of tissue engineering and regenerative medicine. 2007;1(4):261–73. 10.1002/term.37 18038416

[5] Kuo CK, Li WJ, Mauck RL, Tuan RS. Cartilage tissue engineering: its potential and uses. Current opinion in rheumatology. 2006;18(1):64–73. 16344621 10.1097/01.bor.0000198005.88568.df

[6] Nakamura T, Hitomi S, Watanabe S, Shimizu Y, Jamshidi K, Hyon SH, et al Bioabsorption of polylactides with different molecular properties. Journal of biomedical materials research. 1989;23(10):1115–30. 10.1002/jbm.820231003 2808460

[7] Jain RA. The manufacturing techniques of various drug loaded biodegradable poly(lactide-co-glycolide) (PLGA) devices. Biomaterials. 2000;21(23):2475–90. 11055295 10.1016/s0142-9612(00)00115-0

[8] Chang NJ, Lin CC, Li CF, Su K, Yeh ML. The effect of osteochondral regeneration using polymer constructs and continuous passive motion therapy in the lower weight-bearing zone of femoral trocheal groove in rabbits. Annals of biomedical engineering. 2013;41(2):385–97. 10.1007/s10439-012-0656-7 22987137

[9] Chang NJ, Lam CF, Lin CC, Chen WL, Li CF, Lin YT, et al Transplantation of autologous endothelial progenitor cells in porous PLGA scaffolds create a microenvironment for the regeneration of hyaline cartilage in rabbits. Osteoarthritis and cartilage / OARS, Osteoarthritis Research Society. 2013;21(10):1613–22. 10.1016/j.joca.2013.07.016 23927932

[10] Chiang H, Liao CJ, Hsieh CH, Shen CY, Huang YY, Jiang CC. Clinical feasibility of a novel biphasic osteochondral composite for matrix-associated autologous chondrocyte implantation. Osteoarthritis and Cartilage.21(4):589–98. 10.1016/j.joca.2013.01.004 23333470

[11] Frazza EJ, Schmitt EE. A new absorbable suture. Journal of biomedical materials research. 1971;5(2):43–58. 10.1002/jbm.820050207 5575328

[12] Hansen MK, Selnes A, Simonsen E, Sorensen KM, Pedersen GT. [Polyglycolic acid (Dexon) used as suture material for the repair of episiotomies]. Ugeskrift for laeger. 1975;137(11):617–20. 1096390

[13] Shikhman AR, Amiel D, D'Lima D, Hwang SB, Hu C, Xu A, et al Chondroprotective activity of N-acetylglucosamine in rabbits with experimental osteoarthritis. Annals of the rheumatic diseases. 2005;64(1):89–94. 10.1136/ard.2003.019406 15608304 PMC1755188

[14] Namiki O, Toyoshima H, Morisaki N. Therapeutic effect of intra-articular injection of high molecular weight hyaluronic acid on osteoarthritis of the knee. International journal of clinical pharmacology, therapy, and toxicology. 1982;20(11):501–7. 7174151

[15] Sawitzke AD, Shi H, Finco MF, Dunlop DD, Harris CL, Singer NG, et al Clinical efficacy and safety of glucosamine, chondroitin sulphate, their combination, celecoxib or placebo taken to treat osteoarthritis of the knee: 2-year results from GAIT. Annals of the rheumatic diseases. 2010;69(8):1459–64. 10.1136/ard.2009.120469 20525840 PMC3086604

[16] Reginster JY, Deroisy R, Rovati LC, Lee RL, Lejeune E, Bruyere O, et al Long-term effects of glucosamine sulphate on osteoarthritis progression: a randomised, placebo-controlled clinical trial. Lancet. 2001;357(9252):251–6. 10.1016/S0140-6736(00)03610-2 11214126

[17] Pavelka K, Gatterova J, Olejarova M, Machacek S, Giacovelli G, Rovati LC. Glucosamine sulfate use and delay of progression of knee Osteoarthritis—A 3-year, randomized, placebo-controlled, double-blind study. Archives of Internal Medicine. 2002;162(18):2113–23. 12374520 10.1001/archinte.162.18.2113

[18] Sato H, Takahashi T, Ide H, Fukushima T, Tabata M, Sekine F, et al Antioxidant Activity of Synovial-Fluid, Hyaluronic-Acid, and 2 Subcomponents of Hyaluronic-Acid—Synovial-Fluid Scavenging Effect Is Enhanced in Rheumatoid-Arthritis Patients. Arthritis and Rheumatism. 1988;31(1):63–71. 3345232 10.1002/art.1780310110

[19] Shikhman AR, Kuhn K, Alaaeddine N, Lotz M. N-acetylglucosamine prevents IL-1 beta-mediated activation of human chondrocytes. Journal of Immunology. 2001;166(8):5155–60. 10.4049/jimmunol.166.8.5155 11290798

[20] Chang NJ, Lin YT, Lin CC, Wang HC, Hsu HC, Yeh ML. The repair of full-thickness articular cartilage defect using intra-articular administration of N-acetyl-D-glucosamine in the rabbit knee: randomized controlled trial. Biomedical engineering online. 2015;14:105 10.1186/s12938-015-0100-y 26582033 PMC4652361

[21] Jordan KM, Arden NK, Doherty M, Bannwarth B, Bijlsma JW, Dieppe P, et al EULAR Recommendations 2003: an evidence based approach to the management of knee osteoarthritis: Report of a Task Force of the Standing Committee for International Clinical Studies Including Therapeutic Trials (ESCISIT). Annals of the rheumatic diseases. 2003;62(12):1145–55. 10.1136/ard.2003.011742 14644851 PMC1754382

[22] Shim JH, Jang KM, Hahn SK, Park JY, Jung H, Oh K, et al Three-dimensional bioprinting of multilayered constructs containing human mesenchymal stromal cells for osteochondral tissue regeneration in the rabbit knee joint. Biofabrication. 2016;8(1):014102 10.1088/1758-5090/8/1/014102 26844597

[23] Yang J, Liu Y, He L, Wang Q, Wang L, Yuan T, et al Icariin conjugated hyaluronic acid/collagen hydrogel for osteochondral interface restoration. Acta biomaterialia. 2018;74:156–67. 10.1016/j.actbio.2018.05.005 29734010

[24] Henderson IJ, La Valette DP. Subchondral bone overgrowth in the presence of full-thickness cartilage defects in the knee. The Knee. 2005;12(6):435–40. 10.1016/j.knee.2005.04.003 16153850

[25] Minas T, Gomoll AH, Rosenberger R, Royce RO, Bryant T. Increased failure rate of autologous chondrocyte implantation after previous treatment with marrow stimulation techniques. The American journal of sports medicine. 2009;37(5):902–8. 10.1177/0363546508330137 19261905

[26] Chang NJ, Lin CC, Li CF, Wang DA, Issariyaku N, Yeh ML. The combined effects of continuous passive motion treatment and acellular PLGA implants on osteochondral regeneration in the rabbit. Biomaterials. 2012;33(11):3153–63. 10.1016/j.biomaterials.2011.12.054 22264523

[27] Wayne JS, McDowell CL, Shields KJ, Tuan RS. In vivo response of polylactic acid-alginate scaffolds and bone marrow-derived cells for cartilage tissue engineering. Tissue engineering. 2005;11(5–6):953–63. 10.1089/ten.2005.11.953 15998234

[28] O'Driscoll SW, Marx RG, Beaton DE, Miura Y, Gallay SH, Fitzsimmons JS. Validation of a simple histological-histochemical cartilage scoring system. Tissue Eng. 2001;7(3):313–20. 10.1089/10763270152044170 11429151

[29] Jiang YZ, Chen LK, Zhu DC, Zhang GR, Guo C, Qi YY, et al The Inductive Effect of Bone Morphogenetic Protein-4 on Chondral-Lineage Differentiation and In Situ Cartilage Repair. Tissue Eng Pt A. 2010;16(5):1621–32. 10.1089/ten.TEA.2009.0681 20001220

[30] Karin S, Hanna S, Ralf UK, Alexander S, Andreas W, Georg ND, et al Influence of Scaffold Stiffness on Subchondral Bone and Subsequent Cartilage Regeneration in an Ovine Model of Osteochondral Defect Healing. The American journal of sports medicine. 2008;36(12):2379–91. 10.1177/0363546508322899 18952905

[31] Henderson IJP, La Valette DP. Subchondral bone overgrowth in the presence of full-thickness cartilage defects in the knee. The Knee. 2005;12(6):435–40. 10.1016/j.knee.2005.04.003 16153850

[32] Tom M, Andreas HG, Ralf R, Ronald OR, Tim B. Increased Failure Rate of Autologous Chondrocyte Implantation after Previous Treatment with Marrow Stimulation Techniques. The American journal of sports medicine. 2009;37(5):902–8. 10.1177/0363546508330137 19261905

[33] Wu L, Ding J. Effects of porosity and pore size on in vitro degradation of three-dimensional porous poly(D,L-lactide-co-glycolide) scaffolds for tissue engineering. Journal of biomedical materials research Part A. 2005;75(4):767–77. 10.1002/jbm.a.30487 16121386

[34] Chang N-J, Lin C-C, Shie M-Y, Yeh M-L, Li C-F, Liang P-I, et al Positive effects of cell-free porous PLGA implants and early loading exercise on hyaline cartilage regeneration in rabbits. Acta biomaterialia. 2015;28:128–37. 10.1016/j.actbio.2015.09.026 26407650

[35] Nagaoka I, Igarashi M, Sakamoto K. Biological activities of glucosamine and its related substances. Advances in food and nutrition research. 2012;65:337–52. 10.1016/B978-0-12-416003-3.00022-6 22361198

[36] Kothapalli D, Flowers J, Xu T, Pure E, Assoian RK. Differential activation of ERK and Rac mediates the proliferative and anti-proliferative effects of hyaluronan and CD44. The Journal of biological chemistry. 2008;283(46):31823–9. 10.1074/jbc.M802934200 18806267 PMC2581577

[37] Camenisch TD, McDonald JA. Hyaluronan: is bigger better? American journal of respiratory cell and molecular biology. 2000;23(4):431–3. 10.1165/ajrcmb.23.4.f201 11017905

[38] Stern R, Asari AA, Sugahara KN. Hyaluronan fragments: an information-rich system. European journal of cell biology. 2006;85(8):699–715. 10.1016/j.ejcb.2006.05.009 16822580

[39] Feinberg RN, Beebe DC. Hyaluronate in vasculogenesis. Science (New York, NY). 1983;220(4602):1177–9. 10.1126/science.6857242 6857242

[40] Moreland LW. Intra-articular hyaluronan (hyaluronic acid) and hylans for the treatment of osteoarthritis: mechanisms of action. Arthritis research & therapy. 2003;5(2):54–67. 12718745 10.1186/ar623 PMC165033

[41] Miskowiec K, Gadek A, Jurecka A, Sowka J, Slusarski J, Liszka H, et al [Effectiveness and safety of intra-articular use of hyaluronic acid (Suplasyn I-Shot) in the treatment of knee osteoarthritis]. Przeglad lekarski. 2016;73(4):221–3. 27526423

[42] West DC, Kumar S. The effect of hyaluronate and its oligosaccharides on endothelial cell proliferation and monolayer integrity. Experimental cell research. 1989;183(1):179–96. 2472284 10.1016/0014-4827(89)90428-x

[43] Deed R, Rooney P, Kumar P, Norton JD, Smith J, Freemont AJ, et al Early-response gene signalling is induced by angiogenic oligosaccharides of hyaluronan in endothelial cells. Inhibition by non-angiogenic, high-molecular-weight hyaluronan. International journal of cancer. 1997;71(2):251–6. 9139851 10.1002/(sici)1097-0215(19970410)71:2<251::aid-ijc21>3.0.co;2-j

[44] Slevin M, Krupinski J, Kumar S, Gaffney J. Angiogenic oligosaccharides of hyaluronan induce protein tyrosine kinase activity in endothelial cells and activate a cytoplasmic signal transduction pathway resulting in proliferation. Laboratory investigation; a journal of technical methods and pathology. 1998;78(8):987–1003. 9714186

[45] Slevin M, Kumar S, Gaffney J. Angiogenic oligosaccharides of hyaluronan induce multiple signaling pathways affecting vascular endothelial cell mitogenic and wound healing responses. The Journal of biological chemistry. 2002;277(43):41046–59. 10.1074/jbc.M109443200 12194965

[46] Slevin M, West D, Kumar P, Rooney P, Kumar S. Hyaluronan, angiogenesis and malignant disease. International journal of cancer. 2004;109(5):793–4; author reply 5–6. 10.1002/ijc.20059 14999792

[47] Dai J, Rabie AB. VEGF: an essential mediator of both angiogenesis and endochondral ossification. Journal of dental research. 2007;86(10):937–50. 10.1177/154405910708601006 17890669

[48] Arkill KP, Winlove CP. Solute transport in the deep and calcified zones of articular cartilage. Osteoarthritis and cartilage / OARS, Osteoarthritis Research Society. 2008;16(6):708–14. 10.1016/j.joca.2007.10.001 18023368

[49] Pan J, Zhou X, Li W, Novotny JE, Doty SB, Wang L. In situ measurement of transport between subchondral bone and articular cartilage. Journal of orthopaedic research: official publication of the Orthopaedic Research Society. 2009;27(10):1347–52. 10.1002/jor.20883 PMC2748158 19360842

[50] Schagemann JC, Paul S, Casper ME, Rohwedel J, Kramer J, Kaps C, et al Chondrogenic differentiation of bone marrow-derived mesenchymal stromal cells via biomimetic and bioactive poly-epsilon-caprolactone scaffolds. Journal of biomedical materials research Part A. 2013;101(6):1620–8. 10.1002/jbm.a.34457 23184542

[51] Yao H, Xue J, Wang Q, Xie R, Li W, Liu S, et al Glucosamine-modified polyethylene glycol hydrogel-mediated chondrogenic differentiation of human mesenchymal stem cells. Materials science & engineering C, Materials for biological applications. 2017;79:661–70. 28629066 10.1016/j.msec.2017.05.043

[52] Brown TJ, Laurent UB, Fraser JR. Turnover of hyaluronan in synovial joints: elimination of labelled hyaluronan from the knee joint of the rabbit. Experimental physiology. 1991;76(1):125–34. 2015069 10.1113/expphysiol.1991.sp003474

[53] Grimaud E, Heymann D, Redini F. Recent advances in TGF-beta effects on chondrocyte metabolism. Potential therapeutic roles of TGF-beta in cartilage disorders. Cytokine & growth factor reviews. 2002;13(3):241–57. 12486877 10.1016/s1359-6101(02)00004-7

[54] Mendelson A, Frank E, Allred C, Jones E, Chen M, Zhao WL, et al Chondrogenesis by chemotactic homing of synovium, bone marrow, and adipose stem cells in vitro. Faseb J. 2011;25(10):3496–504. 10.1096/fj.10-176305 21746864 PMC3177570

[55] de Haart M, Marijnissen WJCM, van Osch GJVM, Verhaar JAN. Optimization of chondrocyte expansion in culture—Effect of TGF beta-2, bFGF and L-ascorbic acid on bovine articular chondrocytes. Acta Orthop Scand. 1999;70(1):55–61. 10191750 10.3109/17453679909000959

[56] Billinghurst RC, Dahlberg L, Ionescu M, Reiner A, Bourne R, Rorabeck C, et al Enhanced cleavage of type II collagen by collagenases in osteoarthritic articular cartilage. J Clin Invest. 1997;99(7):1534–45. 10.1172/JCI119316 9119997 PMC507973

[57] Huang W, Chung UI, Kronenberg HM, de Crombrugghe B. The chondrogenic transcription factor Sox9 is a target of signaling by the parathyroid hormone-related peptide in the growth plate of endochondral bones. Proceedings of the National Academy of Sciences of the United States of America. 2001;98(1):160–5. 10.1073/pnas.98.1.160 11120880 PMC14561

[58] Oliveira JM, Rodrigues MT, Silva SS, Malafaya PB, Gomes ME, Viegas CA, et al Novel hydroxyapatite/chitosan bilayered scaffold for osteochondral tissue-engineering applications: Scaffold design and its performance when seeded with goat bone marrow stromal cells. Biomaterials. 2006;27(36):6123–37. 10.1016/j.biomaterials.2006.07.034 16945410

[59] Gao J, Dennis JE, Solchaga LA, Awadallah AS, Goldberg VM, Caplan AI. Tissue-engineered fabrication of an osteochondral composite graft using rat bone marrow-derived mesenchymal stem cells. Tissue engineering. 2001;7(4):363–71. 10.1089/10763270152436427 11506726

