# Supplementary figures and images for: Intra-articular injection of N-acetylglucosamine and hyaluronic acid combined with PLGA scaffolds for osteochondral repair in rabbits
Source: PLoS One. 2018 Dec 31;13(12):e0209747. doi: 10.1371/journal.pone.0209747 (PMC6312252; doi:10.1371/journal.pone.0209747)

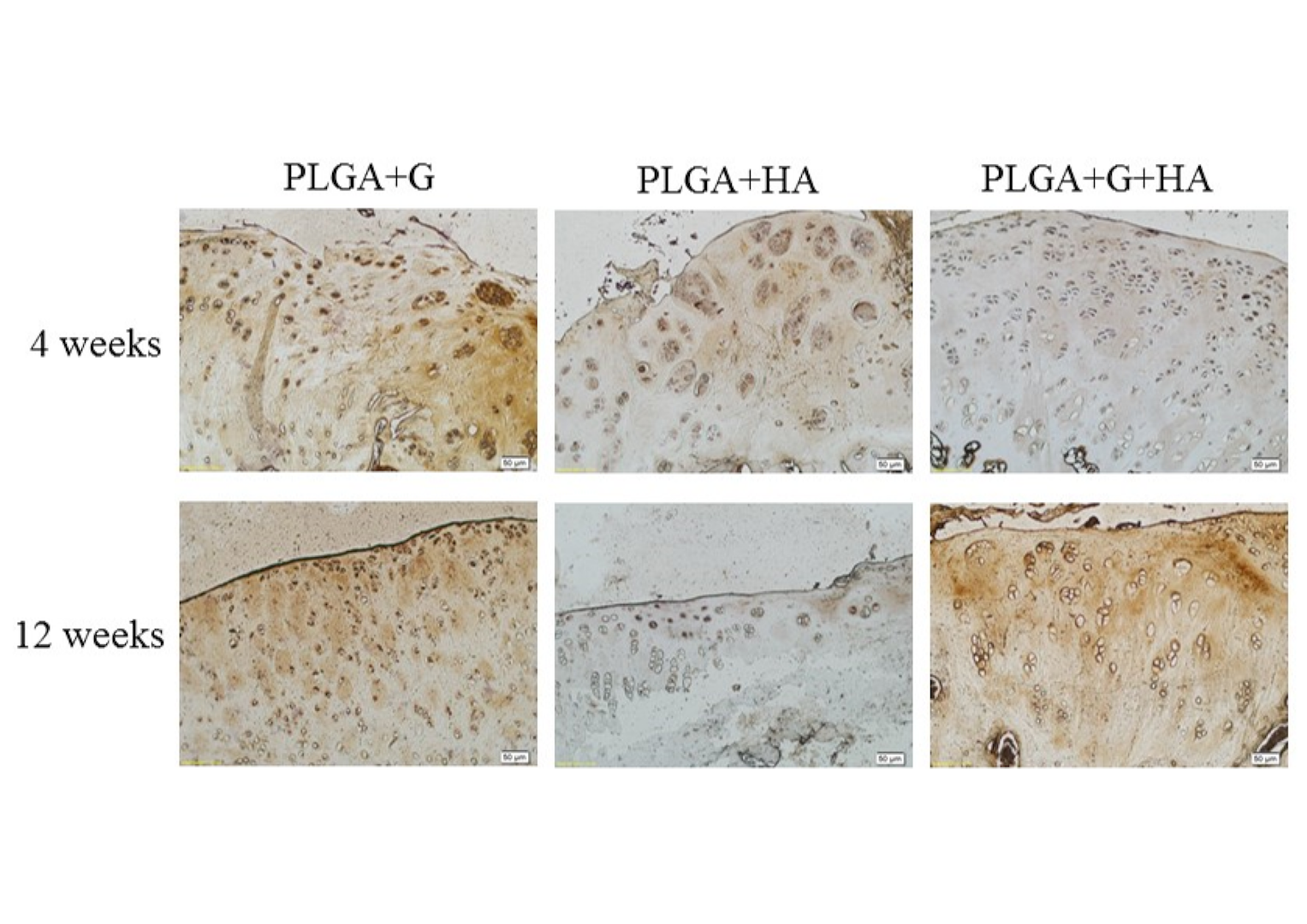

Supplement: S1 Fig — Scale bar: 50 ‘m at 4 weeks and 12 weeks. (TIFF) [file pone.0209747.s001.tiff]

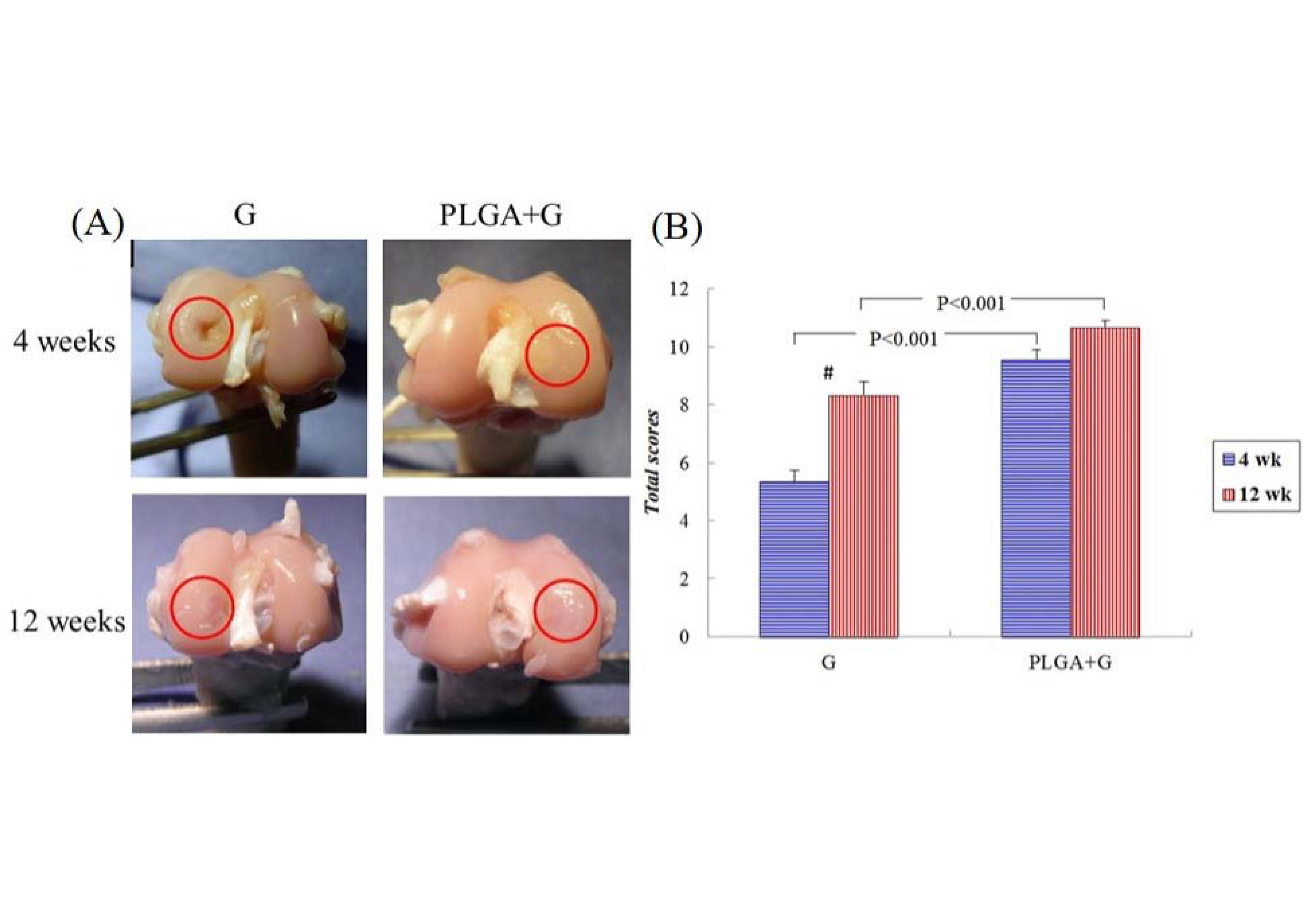

Supplement: S2 Fig — The images of gross appearances in G and PLGA+G groups (A) and Quantitative scores (B) at 4 weeks and 12 weeks after operation. Circles enclose the repaired osteochondral defect area. #: between two time point, p<0.05. (TIF) [file pone.0209747.s002.tif]

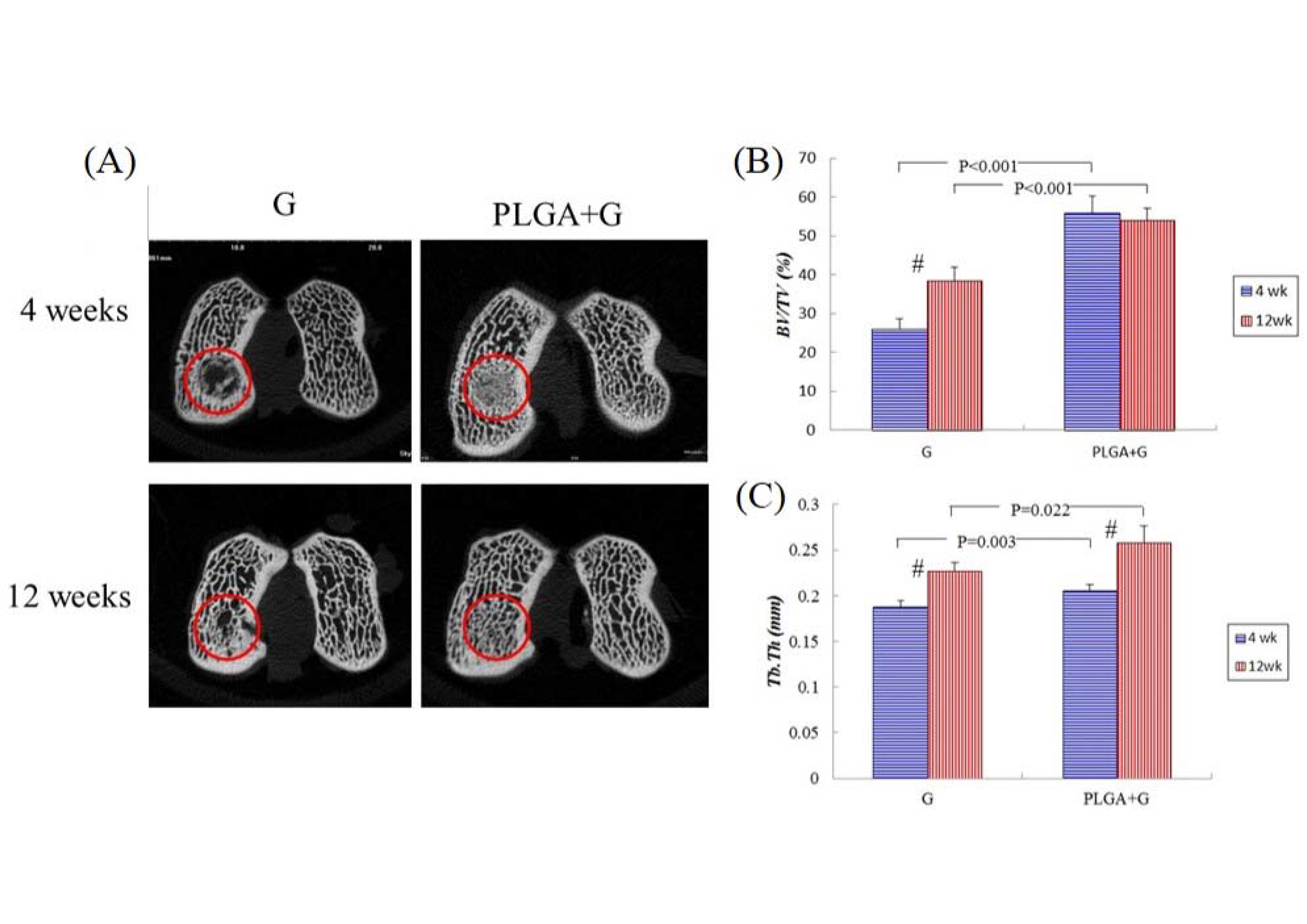

Supplement: S3 Fig — The micro-CT images of bone assessment in G and PLGA+G groups (A) and quantification scores of the TV/BV (B) and Tb.Th (C) at 4 weeks and 12 weeks after operation. Circles enclose the repaired osteochondral defect area. #: between two time point, p<0.05. (TIFF) [file pone.0209747.s003.tiff]
