# Supplementary material for: Intra-articular injection of N-acetylglucosamine and hyaluronic acid combined with PLGA scaffolds for osteochondral repair in rabbits
Source: PLoS One. 2018 Dec 31;13(12):e0209747. doi: 10.1371/journal.pone.0209747 (PMC6312252; doi:10.1371/journal.pone.0209747)
Supplement: S1 Table — (DOCX) [file pone.0209747.s004.docx]

A modified Wayne’s grading scale scoring system for gross appearance

| **Macroscopic Appearance** |  | **Points** |
| --- | --- | --- |
| 1. Coverage | >75% fill  50-75% fill  25-50% fill  <25% fill  0% fill | 4  3  2  1  0 |
| 1. Tissue Color | Normal/whitish  25% yellow/brown/reddish/white  50% yellow/brown/reddish/white  75% yellow/brown/reddish/white  100% yellow/brown/reddish/white | 4  3  2  1  0 |
| 3. Surface (smooth level) | Normal  Smooth but raised  25-50% irregular  50-75% irregular  > 75% irregular | 4  3  2  1  0 |
| *Maximum possible total score* |  | 12 |
